# A Novel Probability-Based Logic-Locking Technique: ProbLock

**DOI:** 10.3390/s21238126

**Published:** 2021-12-04

**Authors:** Michael Yue, Sara Tehranipoor

**Affiliations:** Department of Electrical and Computer Engineering, Santa Clara University, Santa Clara, CA 95053, USA

**Keywords:** hardware security, logic locking, hardware obfuscation

## Abstract

Integrated circuit (IC) piracy and overproduction are serious issues that threaten the security and integrity of a system. Logic locking is a type of hardware obfuscation technique where additional key gates are inserted into the circuit. Only the correct key can unlock the functionality of that circuit; otherwise, the system produces the wrong output. In an effort to hinder these threats on ICs, we have developed a probability-based logic-locking technique to protect the design of a circuit. Our proposed technique, called “ProbLock”, can be applied to both combinational and sequential circuits through a critical selection process. We used a filtering process to select the best location of key gates based on various constraints. Each step in the filtering process generates a subset of nodes for each constraint. We also analyzed the correlation between each constraint and adjusted the strength of the constraints before inserting key gates. We tested our algorithm on 40 benchmarks from the ISCAS ’85 and ISCAS ’89 suites. We evaluated ProbLock against a SAT attack and measured how long the attack took to successfully generate a key value. The SAT attack took longer for most benchmarks using ProbLock which proves viable security in hardware obfuscation.

## 1. Introduction

**Figure 1:**
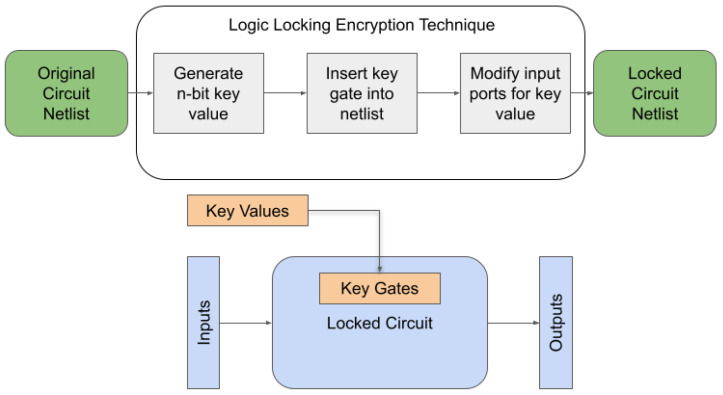
Logic Locking Overview.

**Figure 2:**
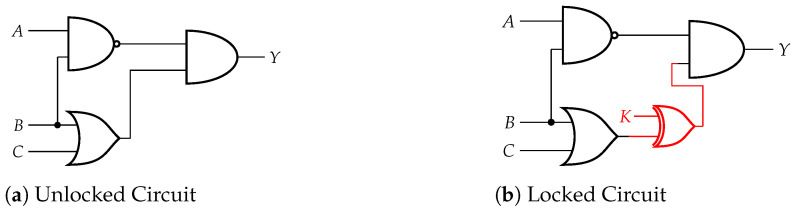
An example of logic locking circuit.

The semiconductor industry is constantly changing from the production of ICs to the complexity of their design. The industry has moved to a fabless model where most of the fabrication for a chip is outsourced to a less secure and less trusted environment. These environments include testing and fabrication facilities that are necessary for the pipeline. While this model improves production costs and development, it has also led to the consequence of piracy, overproduction, and cloning. The chips are also vulnerable to various attacks [1] that attempt to extract the design of the chip or other information from the device. Due to these security issues, researchers have developed techniques to counter these attacks. One technique to improve the security of ICs is hardware obfuscation [2]. Hardware obfuscation is a technique that modifies the structure or description of a circuit in order to make it harder for an attacker to reverse engineer the hardware. Some obfuscation techniques modify the gate level structure of the circuit, while other techniques add gates to protect the logic of the circuit. Logic locking is a technique that inserts additional gates and logic components into a circuit, which then locks the circuit and produces an incorrect output unless the proper key is provided to the circuit. The IC will be considered locked or functionally incorrect until the correct key unlocks the additional gates. Using XOR and XNOR components as key gates, the proper key value will make the gate act as a buffer and have no effect on the rest of the logic. If the wrong key value is provided, the key gate will produce a wrong value and make the circuit nonfunctional. Figure 1 shows an overview of the logic locking technique. The original circuit’s netlist improves with additional key gates and a key-value to unlock the circuit. Figure 2 shows an example of logic locking. A key gate is added between logic gates with one input connected to the key bit value. The addition of these key gates adds a small overhead to the overall circuit while increasing the security of the device.

Various techniques have been proposed by other researchers to protect the integrity and privacy of integrated circuits. Logic cone analysis was used to develop a logic locking technique in 2015 [3]. This technique used fan-in and fan-out metrics to insert key gates into a netlist. Logic cone analysis was vulnerable to SAT attacks that were developed over the next few years. SAT attacks probed the input and output patterns of the system to determine the key to unlock a circuit. SAT attacks have proven to be very effective against logic locking techniques. Strong logic locking (SLL) was another technique developed that analyzed the relationship between inserted key gates in the form of a graph [4,5]. SLL was also vulnerable to SAT-based attacks. A new SAT resistant technique called SARLock was later implemented with the main purpose of thwarting SAT attacks [6]. The method made SAT attacks exponential in complexity and therefore ineffective. In 2019, Tehranipoor et al. [7] explored the potential of employing a state-of-the-art deep RNN that allows an attacker to derive the correct values of the key inputs (secret key) from logic encryption hardware obfuscation techniques.

All of the logic-locking techniques discussed are effective in improving security for an IC; however, they are still vulnerable to sensitization exploits and strong Oracle-based attacks.

To overcome the aforementioned issues, in this paper we propose a very new logic-locking technique that we call “ProbLock”. ProbLock is a probability-based technique that inserts key gates into a circuit netlist where only the correct key value will unlock the circuit to narrows down a set of best nodes to insert key gates. We used four constraints to fit. In this paper, we propose this technique as a form of logic locking where each step of the process narrows the best nodes and chooses the location of the inserted key gates. A probability constraint is the main metric that we used to lock the circuits. We tested our technique by obfuscating a set of circuit benchmarks from ISCAS ’85 and ISCAS ’89 suites [8,9]. These include a variety of combinations and sequential circuits. We analyzed the relationship and correlation between constraints in our technique and found some relationships that support the strength of our technique.

Specifically, this paper makes the following contributions:

- We present a probability-based logic-locking (ProbLock) technique to lock a circuit with low overhead using a filtering process.
- We implemented a design where the strength of the filtering process can be adjusted for different situations.
- We analyzed the correlation between constraints and showed how the relationship between constraints can strengthen the security process.
- We obfuscated 40 benchmarks from ISCAS ’85 and ISCAS ’89 using ProbLock.

### Related Works

Many techniques of logic locking have already been proposed and tested against certain attacks and on circuit benchmarks. One of the earliest logic-locking techniques inserted key gates randomly into the circuit. This provided some security, but many attacks were developed to break this method. Another obfuscation technique was developed using logic-cone analysis in [3]. Sections of a circuit can be grouped into logic cones by calculating the fan-in and fan-out values of a gate. Inserting key gates at certain logic cone areas will increase the security of the system. Logic-cone analysis is good for countering logic-cone attacks. Certain attacks will exploit these weak logic cones and try to discover the key to unlock the circuit. Logic-cone analysis is vulnerable to other types of attacks such as SAT and functional attacks.

Strong logic locking (SLL) is another obfuscation technique, but it is also vulnerable to SAT attacks [4]. SLL is based on interference graphs that show how inserted key gates interfere with each other. The interference graph shows the relationship between an inserted key gate and its surrounding key gates and wires. The interference graph shows if key gates are on a cascading path or parallel path, or if they do not interfere with each other at all. The interference graph along with other information makes it harder for an attacker to unlock the circuit even with SAT attack models. SLL was initially evaluated with a hill-climbing attack where the bits of an initial random key guess is toggled to minimize hamming distance between circuit outputs and test responses. If a key produces a hamming distance of 0, the attack is considered successful. SLL was compared against random logic locking and a fault-based technique. It was shown that the hill-climbing attack was ineffective at determining the correct key value for all tested ISCAS ’85 benchmarks while also being able to break some of the random locked circuits.

More recent techniques have been developed to counter SAT attacks and other related schemes. The obfuscation technique needs to be strong enough to resist certain attacks; otherwise the integrity of the IC would be compromised. The goal of an adversary during an attack is to determine the secret key to unlock the circuit or gain other important information from the system. SARLock was developed to make the SAT attack model inefficient [6]. SARLock employs a small overhead strategy that exponentially increases the number of distinguishing input patterns (DIPs) needed to unlock the circuit. SARLock is very strong against SAT attacks since it uses the basis of the attack model to determine where to insert key gates. The input pattern and corresponding key values can be analyzed during the insertion process of the obfuscation technique. SarLock was evaluated using a SAT attack and calculating the number of DIPs needed to determine the correct key value to unlock a locked circuit. A subset of the ISCAS ’85 benchmarks were encrypted with SarLock and SLL and then evaluated with a SAT attack. SarLock proved that it was more effective against SAT attacks because it took a larger number of DIPs and more time to break the circuit. The SAT algorithm would run for hours to break a SarLock circuit, but it took less than a second for all SLL circuits.

In 2017, TTLock was proposed, which resisted all known attacks including SAT and sensitization attacks [10]. TTLock would invert the response to a logic cone to protect the input pattern. The logic cone would be restored only if the correct key is provided. The small change to the functionality of the circuit would maximize the efforts needed for the SAT attacks. The generalized form of stripping away the functionality of logic cones and hiding it from attackers is known as stripped-functionality logic locking (SFLL). However, the design of the TTLock did not account for the cost of tamper-proof memory, which could lead to high overhead in the re-synthesis process [11,12]. Another group automated the general process of TTLock to identify the parts of the design that needed to be modified in an efficient way. They used ATPG tools to develop a scalable and more efficient way of protecting these patterns from attackers. Overall, a 35% improvement in overhead was achieved with the automated process. Later, a modified version of SFLL was proposed based on the hamming distance of the key. This was referred to as SFLL-hd [13]. The hamming distance metric was used to determine which pattern to modify in the SFLL scheme. Depending on the type of attack, the hamming distance can be adjusted accordingly. In 2019, the idea of exploring high-level synthesis (HLS) with logic locking was proposed with SFLL-HLS [14]. SFLL-HLS was proposed to improve the system-wide security of an IC. The design resulted in faster validation of design and higher levels of abstraction. The HLS implementation in this technique was used to identify the functional units and logic cones to be operated on with respect to SFLL. They observed low overhead and power results from their analysis. The strength of SFLL was evaluated in [13] where a SAT-based attack was developed against SFLL-HLS and other SFLL techniques. Similar to most logic locking techniques, SFLL is vulnerable to strong SAT attacks. The group used synthesized RTL circuits, which were smaller than public benchmark suits from ISCAS ’85 and ISCAS ’89. The SAT attack was able to determine the correct key within seconds for all of these benchmarks. Most recently in 2020, LoPher was developed as another SAT-resistant obfuscation technique [15]. LoPher uses a block cipher to produce the same behavior as a logic gate. The basic component for the block cipher is configurable and allows many logic permutations to occur, which further increases the security of the system.

In 2020, another group presented a scalable attack-resistant obfuscation logic locking technique (SARO) [16]. SARO splits circuit benchmarks into smaller partitions and performs a systematic truth table transformation (T3) for each partition. The T3 process is highly randomized and leads to highly altered structural and graphical representation of the circuit netlist. The first step of SARO is to partition the circuit into a smaller area and analyze the fan in the cone as well as the logic depth of a partition. The fans in cones that cover more inputs are more suitable for the functional transformation of the T3 process. Similarly, a high logic depth allows for more random transformation in structural and graphical representation. Depending on the type of logic gate, selected inputs, and key bit inputs, the T3 process transforms the boolean logic of a gate to add functional obfuscation to the design. The proper key value allows gate logic to function as normally designed. SARO was evaluated against a SAT attack, and for all encrypted ISCAS ’85 benchmarks, the SAT attack was unable to determine the correct key value. SARO is extremely effective against power attack schemes such as a SAT attack; however, it suffers from a high complexity and large overhead. The T3 process has an exponential amount of functions to evaluate, and complexity can vary with number of inputs and key size. The area overhead of SARO is 23%, which is twice as large as most presented obfuscation techniques.

Many forms and variations of SAT attacks have been created in order to show the weaknesses of various hardware obfuscation techniques. Algorithms have been developed for SAT competitions, and the results can be used in a variety of applications including hardware obfuscation [17]. These tools are used to evaluate the strength of logic locking techniques and can be used to bypass the security of integrated circuits. As a result, an anti-SAT unit was developed as a general solution to the SAT attack [18]. The anti-SAT block consists of a low overhead unit that can be added to any obfuscation technique to help counter the SAT attacks. The unit requires the key length for the locked circuit to increase as inputs to the anti-SAT block. The number of DIPs and input patterns that an adversary needs would grow exponentially due to this change. This would make the complexity of the SAT attack exponential instead of linear and therefore inefficient. The recent innovation in anti-SAT has inspired us to develop a technique that will be resistant to various SAT attacks. We designed constraints that should minimize the effects of SAT attack algorithm.

## 2. Materials and Methods

ProbLock is based on filtering out nodes in a circuit to find the best location to insert key gates. ProbLock is a logic-locking technique where the key gates are either XOR or XNOR gates and a key is used to unlock the circuit. We used four constraints to determine the best candidate nodes to insert our XOR or XNOR key gate; **longest path, non-critical path, low dependent nodes, and best probability nodes**. The first three constraints find the set of nodes that lie on the longest path and non-critical path and have low-dependence wires. The last constraint uses probability to find the set of nodes equal to the key length where the key gates will be inserted. We chose the longest path and non-critical path constraint in order to avoid critical timing elements and to insert key gates on parts of the circuit that were being used the most. We chose the low-dependence wires and probability constraint to determine locations where the output would be changed the most. This would make it harder for an attack to generate the golden circuit using an oracle-based attack. Once we determine the location of the key nodes, we can insert key gates into the netlist and re-synthesize the circuit. In Equation (1), the candidate nodes are determined from a function of all four constraints. LP is the set of nodes on the longest paths, while NCP is the set of nodes on non-critical paths. LD represents the set of low-dependence nodes, and *P* are the set of probability nodes.

(1) selectedNodes⊂P⊂LD⊂NCP⊂LP

For our obfuscation technique, we decided to lock a set of combinational and sequential circuit netlists using the ISCAS ’85 and ISCAS ’89 circuit benchmarks. We obfuscated a total of 40 benchmarks using ProbLock. Each of the 40 benchmarks differ in size, so we calculated an appropriate key size for each benchmark and ensured the overhead was less than 10%. The key sizes were generated in powers of 2. We used 8-bit, 16-bit, 32-bit, 64-bit, 128-bit, and 256-bit randomly generated keys to lock differently sized benchmarks. Each bit of a key corresponds to a key gate that adds some overhead to the circuit so that the key size has to be less than 10% of the original circuit to maintain a low overhead. For some of the constraints, we had to use an unrolling technique described in [19] to accurately filter out nodes. This unrolling technique was only used in sequential circuits to simplify the concepts of flip flops and other sequential logics. The sequential logic can be replaced by the main stage and a *k* number of sub-stages depending on the number of times unrolled. This results in a *k*-unrolled circuit that has the same functionality as the regular circuit. For this process, we generated a set of unrolled ISCAS ’89 benchmarks, which we used in some constraint algorithms. We unrolled these circuits once to prevent inaccuracies in constraints such as the longest path and non-critical path.

**Figure 3:**
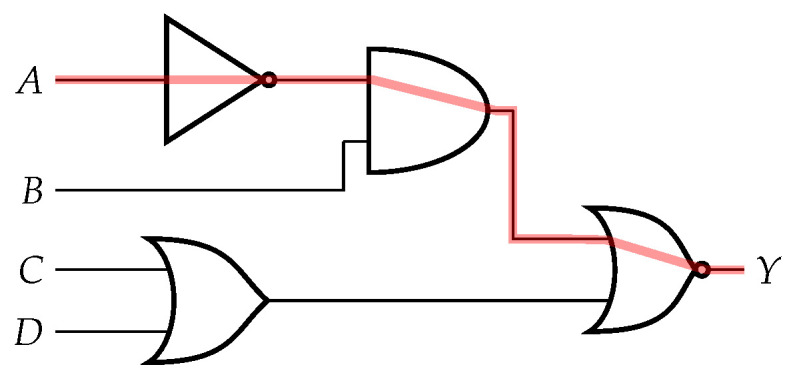
Longest Path (in red).

The longest path constraint isolates a subset of nodes that lie on the longest paths in a circuit netlist. The subset of nodes is different for each circuit and is a function of the key length determined for each circuit. We represent the netlist of each benchmark as a directed acyclic graph (DAG) and perform the longest path analysis on each DAG. Each vertex in the DAG is a gate element from the netlist, and each vector represents the wire connecting to the next gate element. Once the DAG is constructed for each benchmark, we calculated the longest paths of the DAG using a depth first search (DFS) technique. We then calculated the next longest path to generate a subset of nodes along the longest paths. Each unique node in the longest path is added to a subset during each iteration until the size of the subset is larger than two times the key length for that circuit. The structure of this theory is shown in Algorithm 1, which uses the DFS in Algorithm 2. Figure 3 shows the longest path for the circuit to be 3 since there are 3 gates between input *A* and output *Y*. The next longest path would also be 3 from input *B* to output *Y*. All of the nodes along both longest paths would be added to a subset of the longest path nodes. Once this subset of longest path nodes is determined, that subset is used in the next filtering constraint. This subset can be adjusted to include more or fewer nodes depending on other filtering constraints. If more nodes are needed, this constraint is the first to be modified.

We chose to use the longest path constraint in order to counter oracle-guided attacks. Oracle-guided attacks will query the IC with various inputs and observe the output. This gives the attacker information about how the circuit behaves, and the adversary can use this information to determine the secret key. We want to insert key gates where most of the logic and activity occur in the circuit. An oracle-guided attack will most likely pass data through the longest paths of a circuit, so we want to protect these parts of the IC by inserting key gates on the longest path.
**Algorithm 1:** Get Longest Path.
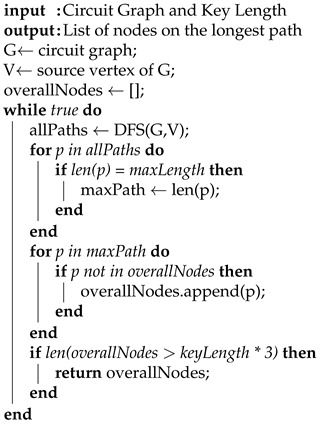

**Algorithm 2:** Depth First Search.
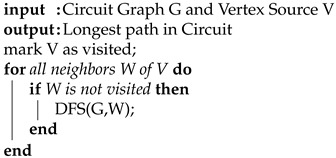


**Figure 4:**
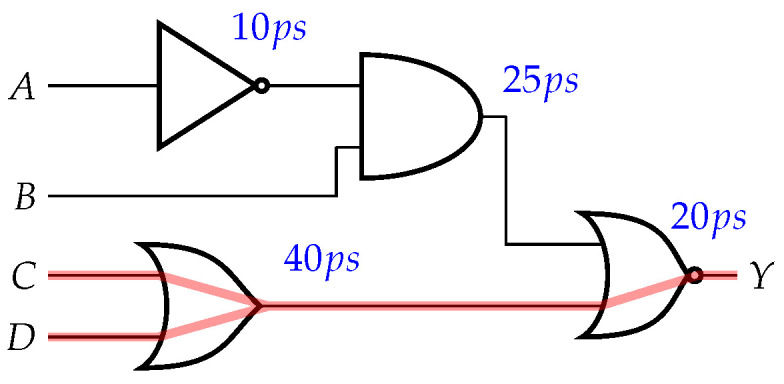
Critical paths (in red).

The critical path constraint is similar to the longest path; however, rather than considering logic depth, we look at timing information. As shown in Figure 4, the critical paths are shown in red. This constraint is essential, as adding gates on the critical path could break the circuit functionality or change timing specifications. The nodes selected often overlapped with other constraints (e.g., the longest path was often the critical path), though oftentimes the critical path would involve gates with large fan-out. Determining the critical path is largely technology-specific; different PDKs will have different timing information, which can affect which paths are critical paths. We removed any nodes that were on the critical path from the set of nodes passed into this constraint. The resulting subset results in nodes that are on the longest, non-critical path.

The next constraint generates a subset of nodes that are connected to low-dependence wires. The output wire of a gate is considered low dependence if the input wires to that gate have little influence on the value of output. This idea is modified from a technique called FANCI where suspicious wires can be detected in a Trojan-infected design [20]. A functional truth table is created for each output wire of each gate in the circuit. The inputs of the truth table correspond to the inputs of the gate being analyzed. For each input column, the other columns are fixed and each row is tested with a 0 or 1 to determine the output. This results in two functions when setting the value to either 0 or 1. The boolean difference between these two functions results in a value between zero and one that can be further analyzed. The value for each input gets stored as a list for each output wire. We take the average value of the entire list to determine the dependency of an output wire. The algorithm logic is shown in Figure 3. This analysis can determine if certain inputs are low dependence or if they rarely affect the corresponding logic. Low-dependent wires are weak spots in the circuit so this constraint isolates those locations in order to improve security. We insert key gates next to low-dependence wires to fortify any weaknesses. The filtering process passes the subset of nodes to the final constraint.

The probability constraint focuses on reducing the effectiveness of the SAT attacks. In a SAT attack, a distinguishing input (DI) is chosen and the attacker runs through various key values, eliminating any that yield an incorrect output. Thus, to reduce the effectiveness of a SAT attack, the number of wrong keys produced for a given DI must decrease. This can be done by bringing the probability of any given node being 1 closer to 0.5, since any node that is biased towards 0 or 1 will propagate through to the output nodes, making it easier for SAT attacks to eliminate key values. Since a two-input XOR/XNOR has an output probability of 0.5, we can insert our key gates at nodes heavily biased towards 0 or 1 and “reset” the probability to 0.5.

The algorithm used to obtain the *N* nodes with the most biased probabilities is shown in Algorithm 4. It is worth noting that while generating node probabilities for combinational circuits is trivial, sequential circuits pose a potential problem because of the D flip flops (DFFs). However, giving the DFF outputs a starting probability of 0.5 and propagating running a few iterations (three is sufficient) will asymptotically approach the correct probability for the DFF node.
**Algorithm 3:** Find Low Dependent Wires.
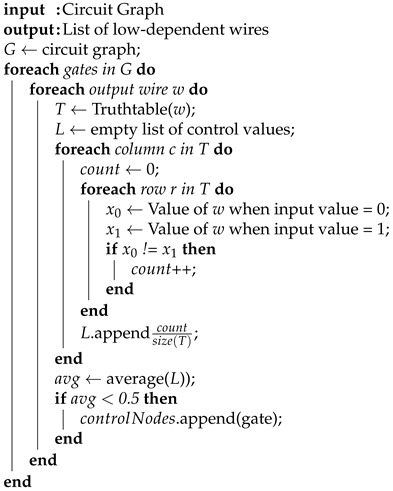

**Algorithm 4:** Find Biased Nodes.
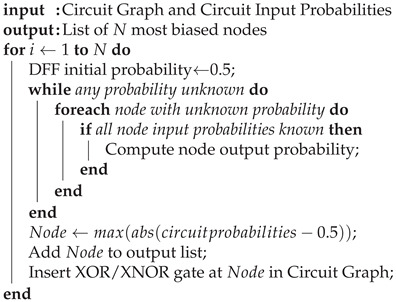


**Figure 5:**
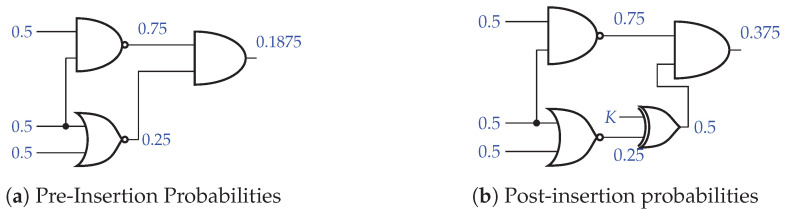
Key gate insertion probabilities.

An example of this is illustrated in Figure 5. Figure 5a shows a sample circuit with each node annotated with the probability of that node being a logic 1. The output shown is heavily biased toward logic 0, which makes it more susceptible to SAT attacks. Strategically adding a key gate, as shown in Figure 5b, brings the output probability closer to 0.5, reducing the effectiveness of the SAT attack.

To obfuscate the benchmarks, we created a python script that implements this algorithm. The script takes in a benchmark netlist in Verilog format and returns an obfuscated netlist in the same format. The obfuscated netlist included the key gates inserted as well as the key defined to unlock the circuit. We created a function to parse each netlist for information. The information was organized into lists of inputs, outputs, and gate types. We used this information to determine the key size relative to the number of gates in a netlist. We also created functions for each constraint in our algorithm. A set of overall nodes was passed through each function and then narrowed down to a set of best nodes for key gate insertion. Another function was created to insert key gates from a data structure into a new netlist. We specified the key inputs, key gates, and the key value in the header of the new netlist for development purposes. Throughout the development process, we ran tests to verify the intention of our script and to make sure each new netlist was correct.

We used Synopsys Design Compiler to synthesize and view the netlists before and after obfuscation [21]. The Synopsys tool allowed us to see the gate-level representation of each benchmark during the analysis process. We were able to see the location of the logic components as well as the inserted key gate after the obfuscation process occurred. We also used the Design Compiler for critical path analysis in our second constraint. The tool allowed for timing analysis between different logic components of the netlist. We used this to calculate the critical paths for our constraint and removed any nodes that lie on this critical path. We integrated the results from Synopsys by passing them through a text file that gets parsed in the main script.

## 3. Experimental Results

During the development process of ProbLock, we analyzed the correlation and relationship between constraints. We documented this relationship to show how each constraint impacted the overall strength of the technique. We chose two constraints based on path elements and two constraints based on nodes and wires. Due to this design, we were able to analyze the correlation between constraints and adjust the strength of the filtering process based on this analysis. Overall, we wanted the correlation between constraints to be large enough to remove any nodes that did not belong in both sets. This would allow the filtering process from each constraint to generate a subset of nodes each time until only the best candidate nodes remained to be inserted. The strength of the correlation varies between benchmarks because of the shape and functionality of each circuit. Each subsequent constraint filtered out a set of nodes based on the relationship between the constraint and the overall set of nodes. Table 1, Table 2, Table 3 and Table 4 show the experimental correlations for ISCAS ’85 and ’89 benchmarks. We only show some of the results in the table as all 40 benchmarks are not included. The longest path (LP) length and critical path (CP) count are based on the path constraints. The rest of the categories including non-critical path (NCP), low depending wires (LD), and biased probabilities (Prob) are based on nodes.

For each constraint, a smaller set of nodes is generated until the final set is determined, which corresponds to the location of inserted key gates. In the ISCAS ’85 benchmark suite, the correlation between nodes on the longest path and the original set of nodes was 36% on average. Between nodes on the non-critical path and nodes on the longest path, the correlation was about 63% on average. The correlation between low-dependence nodes and nodes on the non-critical path was about 73% and the final correlation between biased probabilities and low-dependence nodes was about 65%. For the ISCAS ’89 benchmark suite, the correlation between the longest path and overall nodes is 27%. The correlation between the critical path and the longest path is 84%. The correlation between low-dependence nodes and the non-critical path is 85%, and the final correlation between biased probabilities and low-dependence nodes is 45%. The numbers that we analyzed were ideal for the filtering process. Enough nodes were removed with each subset until the final set of best candidates was discovered. For the final biased probabilities constraint, the final set of nodes was equal to the size of the key. For the other constraints, we adjusted the filtering threshold accordingly. Depending on the situation, the strength of the constraints can be adjusted, which allows flexibility in our algorithm. After re-synthesizing the obfuscated netlists, we used Synopsys to verify the behavior of the locked circuits [21]. The critical path of the netlists remained the same, and the timing analysis remained consistent for all benchmarks. The non-critical path constraint was important to ensure that the timing components of the circuit were not affected. We also used Synopsys to verify that the overhead of netlist was no greater than 10%. Most of the circuits had an overhead around 7%, but the larger benchmarks had less than 5% overhead due to 256-bit key being the largest key size for benchmarks with thousands of gates.

We tested ProbLock against other encryption techniques from similar logic-locking papers. We measured the decryption time against a SAT attack algorithm and compared the metric to evaluate security effectiveness. The SAT algorithm from [22] performs a basic SAT attack on locked logic circuits. The attack is successful if the correct key can be determined in an appropriate amount of time. If a key value can be determined, the length of the attack is measured for analysis. The longer decryption time correlates to stronger security for the encryption technique. The first two techniques we tested were toc13xor and dac12 [22], which are standard XOR/XNOR insertion techniques based on maximizing hamming distance and minimizing the effectiveness of a fault analysis attack. The set of benchmarks available included ISCAS ’85 combinational benchmarks. The next technique we included in the study was a logic cone key gate insertion technique (LCSB) [12]. Finally, we tested the effectiveness of ProbLock by comparing the decryption time from a SAT attack for all four encryption techniques. Toc13xor and dac12 were provided with an encryption script so we could generate a set of locked circuits with different key values. The LCSB algorithm was implemented separately since it was an easy algorithm to use. For each benchmark, ten locked circuits were generated for each encryption technique and evaluated using a SAT solver. The average encryption times were recorded in Table 5 and Table 6. The results show that ProbLock is more secure than the other techniques for the majority of the combinational benchmarks. The overhead is also comparable for all circuits evaluated. Each technique that we used to lock a circuit, including ProbLock, had less than 10% area overhead. We also encrypted the ISCAS ’89 benchmarks with ProbLock and evaluated the circuits against the same SAT attack. Since we did not have access to ISCAS ’89 netlists using other obfuscation techniques, we could not properly compare and analyze our results with an appropriate baseline.

## 4. Discussion

As shown by the results, ProbLock is a good logic-locking encryption technique. The probability constraint presented by ProbLock is a novel metric used to develop a new logic locking technique. For most of the combinational benchmarks, ProbLock has a longer decryption time and better security. The experiment can be improved and ProbLock itself can be optimized for better results. Some of the encryption benchmarks provided to us from previous studies had missing circuits so we could not compare them in the experiment. We also only tested combinational circuits even though ProbLock could be used on sequential circuits. Since toc13xor, dac12, and LCSB did not work on sequential circuits, we did not have enough data to compare against ProbLock. This will be a focus point for further exploration while we improve ProbLock. The area overhead of ProbLock also proves the simplicity of the algorithm. Only one additional logic gate is required for each bit of the key to lock a circuit. This allows for a very small overhead while gaining maximum security with the obfuscation technique. A low overhead also allows for future combination of other methods along with ProbLock. For example, encryption-independent anti-SAT blocks can be integrated with ProbLock to improve security while continuing to have a low area overhead [18].

Some of the limitations of ProbLock include the high complexity and lack of experimental data needed for comparison. For each iteration of adding a new key gate, the probability metric of each gate changes. The algorithm for the probability constraint is performed *k* times for each bit of the key. This adds a large amount of computation time that can be improved and optimized in later efforts. Most of the algorithms used to support ProbLock can also be optimized for a lower algorithmic complexity. Another way to improve this study is to use the data set used by other similar studies. The ISCAS ’85 dataset is a publicly available combinational circuit benchmark that has been used by many studies in the field of hardware security. The ISCAS ’89 suite is a similar set of sequential benchmarks but it is harder to work with due to the sequential logic. We did not do a conclusive study on the sequential circuits because there were not enough prior data to do a comparison.

Compared to other logic-locking studies, ProbLock uses the same approach and evaluation methods. We encrypted a set of publicly available benchmarks using our technique and evaluated the strength of the obfuscation using an attack algorithm to determine the correct key value needed to unlock the circuit. Similar to other studies, we used ISCAS ’85 and ISCAS ’89 as our benchmark data. Other studies used less available benchmarks along with the combination suite of ISCAS ’85, which is why our results are only valid for ISCAS ’85 and not ISCAS ’89. Other studies also rarely evaluated their technique against a SAT solver. SAT solvers are extremely powerful and often can break simple hardware obfuscation techniques. For studies that are effective against SAT attacks such as [16], higher overhead and complexity are needed to make the encryption stronger. For obfuscation techniques that employ the same level of overhead and complexity such as SLL and LCSB [4,12], ProbLock had better results against a stronger attack.

## 5. Conclusions and Future Works

In this paper, we propose the ProbLock method that is a probability-based logic locking technique and uses a filtering process to determine the location of inserted key gates. ProbLock uses four constraints to narrow the set of nodes in a netlist to be used for insertion. We obfuscated 40 different sequential and combinational benchmarks from the ISCAS ’85 and ISCAS ’89 suite. After obfuscating the circuits, we analyzed the correlation between constraints and implemented the capability to adjust these constraints depending on the situation. In the future, we intend to test the obfuscated benchmarks against known attacks and compare them to other logic locking techniques. We will implement logic locking attacks such as SAT attacks and sensitization attacks. Each attack will be executed against benchmarks obfuscated with ProbLock. We will then run the same attacks on locking schemes such as SLL [4], logic cone locking [3], and SARLock [6]. We will evaluate how well each benchmark performs by measuring the overhead of the obfuscation technique, complexity of the technique, and execution time of the attack. After running each attack scheme, we can compare and evaluate the true strength of ProbLock compared to other published logic-locking techniques.

We also plan on strengthening ProbLock against SAT attacks by integrating SAT-resistant logic near the key gate locations. This would increase overhead, but we would also try to optimize this in our experiment. After an initial analysis of integrating an anti-SAT block into ProbLock, we determined that we can reuse some of the key gates from ProbLock into building an anti-SAT block to further thwart the effectiveness of a SAT attack. We would use existing key gates along with additional logic to a build a module that would exponentially increase the number of iterations in the SAT attack algorithm. Currently, the overhead for this extension is less than 18%. We would conduct a similar experiment with the new integration of the anti-SAT block and evaluate the execution time against the same SAT solver.

## Figures and Tables

**Table 1:** ISCAS ’85 Node Count.

ISCAS 85	Key Size	LP Length	CP Count
c432	16	18	7
c499	16	12	32
c1355	32	25	32
c1908	64	39	25
c2670	64	31	100
c3540	128	42	22
c5315	128	47	100
c7552	256	35	100

**Table 2:** ISCAS ’85 Constraint Correlation.

ISCAS 85	Total Nodes	LP Subset	NCP Subset	LD Subset	Prob Subset
c432	160	88	60	33	16
c499	202	186	104	99	16
c1355	546	485	253	53	32
c1908	880	205	145	129	64
c2670	1269	217	200	75	64
c3540	1669	260	173	151	128
c5315	2307	411	226	180	128
c7552	3513	532	341	278	256

**Table 3:** ISCAS ’89 Node Count.

ISCAS 89	Key Size	LP Length	CP Count
s298	8	10	6
s344	8	21	11
s382	8	10	6
s386	8	12	7
s400	8	10	6
s444	8	12	6
s526	8	10	6
s641	8	75	24
s713	8	75	23
s838	16	18	1
s1238a	32	23	14
s1488	32	18	19
s5378a	64	15	46
s9234a	128	19	37
s13207a	256	28	100
s15850a	256	22	100
s38584	256	15	100

**Table 4:** ISCAS ’89 Constraint Correlation.

ISCAS 89	Total Nodes	LP Subset	NCP subset	LD Subset	Prob Subset
s298	75	26	26	18	8
s344	101	21	19	19	8
s382	99	29	29	21	8
s386	118	49	32	24	8
s400	106	30	30	22	8
s444	119	38	38	30	8
s526	141	26	26	18	8
s641	107	80	53	51	8
s713	139	84	61	56	8
s838	288	45	29	26	16
s1238a	428	132	76	64	32
s1488	550	89	60	48	32
s5378a	1004	134	96	92	64
s9234a	2027	264	261	213	128
s13207a	2573	573	521	482	256
s15850a	3448	553	544	506	256
s38584	11,448	717	716	571	256

**Table 5:** ISCAS ’85 SAT Attack Results.

ISCAS 85	Encryption	Decryption Time (s)	Encryption	Decryption Time (s)
c17	toc13xor	n/a	dac12	n/a
c432	toc13xor	0.057259	dac12	0.051281
c499	toc13xor	0.1015	dac12	0.115953
c880a	toc13xor	0.144556	dac12	0.157413
c1355	toc13xor	0.188653	dac12	0.393672
c1908	toc13xor	3.93094	dac12	0.673176
c2670	toc13xor	0.285035	dac12	unbreakable
c3540	toc13xor	1.20809	dac12	3.51718
c5315	toc13xor	0.947552	dac12	9.74864
c6288	toc13xor	n/a	dac12	n/a
c7552	toc13xor	1.51212	dac12	28.5707

**Table 6:** ISCAS ’85 SAT Attack Results.

ISCAS 85	Encryption	Decryption Time (s)	Encryption	Decryption Time (s)
c17	LCSB	0.015533	ProbLock	0.005433
c432	LCSB	0.05647	ProbLock	0.10256
c499	LCSB	0.116477	ProbLock	0.2273
c880a	LCSB	0.178235	ProbLock	1.6596
c1355	LCSB	0.05869	ProbLock	unbreakable
c1908	LCSB	2.66502	ProbLock	2.0384
c2670	LCSB	2.2984	ProbLock	145.8
c3540	LCSB	1.51982	ProbLock	8.291
c5315	LCSB	4.65455	ProbLock	94.024
c6288	LCSB	6.01653	ProbLock	1.2284
c7552	LCSB	3.08791	ProbLock	674.5

## Data Availability

The ISCAS ’85 and ISCAS ’89 benchmarks that are used in this study are openly available online [8,9]. The full suite of benchmarks are a set of combinational and sequential logic circuits that can be used for testing and simulation. ISCAS benchmarks were developed by Franc Brglez for research purposes.
